# Eu^2+^ and Eu^3+^ Doubly Doped ZnWO_4_ Nanoplates with Superior Photocatalytic Performance for Dye Degradation

**DOI:** 10.3390/nano8100765

**Published:** 2018-09-27

**Authors:** Yuan Ming Huang, Ming Yu Li, Long Yang, Bao-gai Zhai

**Affiliations:** School of Mathematics and Physics, Changzhou University, Changzhou 213164, China; ymhuang@cczu.edu.cn (Y.M.H.); 16106108@smail.cczu.edu.cn (M.Y.L.); 16106105@smail.cczu.edu.cn (L.Y.)

**Keywords:** ZnWO_4_ nanoplates, facet engineering, Eu doping, photocatalytic performance, hydrothermal method

## Abstract

Eu^2+^ and Eu^3+^ doubly doped ZnWO_4_ nanoplates with highly exposed {100} facets were synthesized via a facile hydrothermal route in the presence of surfactant cetyltrimethyl ammonium bromide. These ZnWO_4_ nanoplates were characterized using scanning electron microscopy, transmission electron microscopy, X-ray diffraction, X-ray photoelectron spectrometry, diffuse UV-vis reflectance spectroscopy, photoluminescence spectrophotometry, and photoluminescence lifetime spectroscopy to determine their morphological, structural, chemical, and optical characteristics. It is found that Eu-doped ZnWO_4_ nanoplates exhibit superior photo-oxidative capability to completely mineralize the methyl orange into CO_2_ and H_2_O, whereas undoped ZnWO_4_ nanoparticles can only cleave the organic molecules into fragments. The superior photocatalytic performance of Eu-doped ZnWO_4_ nanoplates can be attributed to the cooperative effects of crystal facet engineering and defect engineering. This is a valuable report on crystal facet engineering in combination with defect engineering for the development of highly efficient photocatalysts.

## 1. Introduction

Belonging to a wide group of wolframite-type tungstates, zinc tungstate (ZnWO_4_) is a technologically important material for numerous applications in the fields of luminescent materials [1,2,3,4,5], photocatalysts [6,7,8,9,10,11,12,13,14], Li-ion batteries [15], humidity sensors [16], materials for stimulated Raman scattering [17], and materials for deactivating microorganisms [18]. Among these applications, the photocatalytic properties of ZnWO_4_ nanostructures have been intensively investigated in order to solve one of the most serious environmental problems in our modern society via semiconductor-based photocatalytic degradation of organic contaminants in water under sunlight [6,7,8,9,10,11]. Up to date, a diverse range of strategies has been developed to enhance the photocatalytic activity of ZnWO_4_ nanostructures, which can be classified into three categories: (i) synthesis of ZnWO_4_ nanorods and nanosheets with large specific surface area [19]; (ii) coupling ZnWO_4_ with other semiconductors and metals such as In_2_S_3_ [20], Ag [21], ZnO [22], and Cu_2_O [23]; and (iii) defect engineering ZnWO_4_ via doping with non-metal ions (B, C, N, F) [24,25,26], transition metal ions (Sn^2+^, Cr^3+^, Mn^2+^, Cu^2+^) [27,28], and lanthanide ions (Dy^3+^, Er^3+^) [29,30]. Interestingly, the defect engineering is found to be able to significantly enhance the photocatalytic performances of ZnWO_4_ nanostructures. For example, Phuruangrat et al. reported that the activity of Dy^3+^ doped ZnWO_4_ nanorods (3 mol %) was 1.5 times of that of undoped ZnWO_4_ [29]; Zhou et al. observed the enhanced photocatalytic activity of Er^3+^ doped ZnWO_4_ nanorods [30].

Besides the above mentioned strategies, crystal facet engineering has recently become an important technique to improve the photocatalytic activity of semiconductor-based photocatalysts [31,32,33,34,35]. It is known that crystal facet engineering of a semiconductor photocatalyst can induce exotic physical and chemical performance in the photocatalyst because of the differently exposed ions on the different facets. Previous explorations have shown the profound influence of facets on the photocatalysis. For example, Yuan et al. demonstrated the crystal facet-correlated photocatalytic activity of α-Fe_2_O_3_ for water splitting [35]; Wu et al. reported that {010} faceted BiOBr nanocrystals displayed a better photo-oxidative capability than {001} faceted nanocrystals for water oxidation and formic acid degradation [36]; Qi et al. found that {101} faceted TiO_2_ showed superior catalytic activity to {001} and {010} faceted TiO_2_ for anthracene degradation [37]; Rong et al. reported that {310} faceted α-MnO_2_ nanowires exhibited much better activity than {100} and {110} facets for formaldehyde oxidation [38]. It is clear that little attention is paid on the facet-dependent photocatalytic activity of ZnWO_4_, although such a phenomenon is well studied in a number of functional materials [31,32,33,34,35,36,37,38].

By combining the defect engineering (doping) with facet engineering, we anticipate that Eu-doped ZnWO_4_ nanoplates with highly exposed {100} facets might have noticeably different photocatalytic performance when compared with ZnWO_4_ nanoparticles, which have no clearly defined facets. In this work, Eu-doped ZnWO_4_ nanoplates with highly exposed {100} facets were synthesized via the hydrothermal technique. When compared with undoped ZnWO_4_ nanoparticles, we demonstrated that the Eu-doped ZnWO_4_ nanoplates exhibit superior photo-oxidative capability to completely mineralize dye molecules into CO_2_ and H_2_O, whereas the undoped ZnWO_4_ nanoparticles cannot do so. This work provides new insights into the development of highly efficient photocatalysts for pollutant elimination through crystal-facet tailoring in combination with defect-engineering. To our knowledge, there is hardly any report about lanthanide ions doubly doped ZnWO_4_ nanoplates with highly exposed facets for highly efficient photocatalyst.

## 2. Materials and Characterizations

### 2.1. Preparation of Eu-Doped ZnWO_4_ Nanoplates

Eu-doped ZnWO_4_ nanoplates were prepared via the hydrothermal route. Analytical grade reagents Na_2_WO_4_·2H_2_O, Zn(NO_3_)_2_·6H_2_O, Eu(NO_3_)·6H_2_O, cetyltrimethyl ammonium bromide (CTAB), and ammonia were provided by Sinopharm Chemical Reagents Company (Shanghai, China). Under vigorous stirring with a magnetic bar, Zn(NO_3_)_2_·6H_2_O (0.01 mol), CTAB (0.001 mol), Eu(NO_3_)·6H_2_O (0.0005 mol), and Na_2_WO_4_·2H_2_O (0.01 mol) were dissolved into 80 mL deionized water. The pH value of the reaction system was adjusted to around 9 by adding appropriate amount of ammonia. After stirring for 30 min, the mixture was transferred into a 90 mL Teflon-lined stainless steel autoclave. With a filling capacity of about 90%, the autoclave was sealed and maintained at 180 °C for 17 h for hydrothermal reaction. After being cooled to room temperature in air, the precipitates from the autoclave were filtered, washed repeatedly with deionized water, and then dried in an oven at 90 °C for 6 h. In the process of hydrothermal synthesis, a portion of Eu^3+^ ions were reduced to Eu^2+^, but the total molar concentration of Eu^2+^ and Eu^3+^ in ZnWO_4_ was fixed to be 5 mol % [39]. Undoped ZnWO_4_ nanoparticles, which have no well defined facets, were employed as a reference photocatalyst. Without the addition of Eu(NO_3_)·6H_2_O into the starting materials, undoped ZnWO_4_ nanoparticles were prepared via the hydrothermal route under the condition of pH = 5.65, while the other parameters were kept unchanged.

### 2.2. Crystal Structure and Morphology of Eu-Doped ZnWO_4_ Nanoplates

The scanning electron microscope (SEM) (S-4800, Hitachi, Tokyo, Japan) and X-ray diffractometer (XRD) (D/max 2500 PC, Akishima, Japan) were employed to analyze the morphology and crystal structures of the synthesized ZnWO_4_ nanoplates. The SEM was coupled with a silicon drifted detector as the X-ray analyzer for the energy dispersive X-ray (EDX) spectroscopic analysis. The nanostructures of the sample were characterized on a transmission electron microscope (TEM) (JEOL JEM–2100, Japan Electronics Corp. Akishima, Japan), which was operated at 200 kV. The X-ray photoelectron spectroscopic (XPS) measurements were performed on an Escalab 250Xi spectrophotometer (Thermo Scientific, Waltham, MA, USA) with Al Kα radiation (1486.6 eV). The XPS spectrometer was calibrated by recording the binding energy of Au4f_7/2_ peak at 83.9 eV. A C1s peak at 284.6 eV was taken as an internal standard.

### 2.3. Absorption and PL Spectra of Eu-Doped ZnWO_4_ Nanoplates

The diffused reflectance spectra of the samples were measured with a UV-vis spectrometer (UV3600, Shimazu, Kyoto, Japan). The photoluminescence (PL) spectra of ZnWO_4_ nanoplates were recorded with a spectrophotometer (Tianjin Gangdong Ltd., Tianjin, China). The 325 nm laser line from a helium-cadmium laser was utilized as the excitation source for the PL measurement. The PL lifetime spectra of the ZnWO_4_ nanoplates were measured at room temperature on a picosecond fluorescence lifetime spectrometer (LifeSpec II, Edinburgh Instruments, Edinburgh, UK), utilizing a time correlated single photon counting method with a pulsed diode laser source (λ = 375 nm). The typical pulse width, peak power, and repetition frequency of the picosecond pulsed diode laser were 50 ps, 90 mW, and 20 MHz, respectively. Details on the characterizations could be found elsewhere [40,41].

### 2.4. Electronic Structure Calculation of ZnWO_4_

First-principles density functional theory (DFT) calculations of the electronic structures of ZnWO_4_ were performed using the DFT module of the Quantumwise Atomistix ToolKit 11.8 package. The exchange-correlation functional was treated within the GGA + U scheme, in which GGA was described by the Perdew–Burke–Ernzerhof potential [42], whereas U^2p^ = 0 eV for O, U^5d^ = 8 eV for W, and U^3d^ = 0 eV for Zn. Monoclinic ZnWO_4_ belongs to space group P2/c (13). There are 2 Zn, 2 W, and 8 O atoms in the unit cell of ZnWO_4_. The initial structural data of ZnWO_4_ were taken from Inorganic Crystal Structure Database (ICSD No. 156483). The lattice parameters of monoclinic ZnWO_4_ were taken as *a =* 0.4691 nm, *b* = 0.572 nm, *c* = 0.4925 nm, and *β* = 90.64° in the present work. The considered electronic configurations were 3d^10^4p^0^4s^2^ for Zn, 2s^2^2p^4^ for O, and 5p^6^5d^4^6s^2^ for W. Double zeta single polarized basis sets were chosen for each element. The electronic wave-functions were expanded in plane waves up to a kinetic energy cut-off with a typical value of 100 Hartree. The Monkhorst–Pack scheme *k*-points grid sampling was set at 5 × 5 × 5 for the Brillouin zone. The Brillouin zone sampling and the kinetic energy cutoff were sufficient to guarantee an excellent convergence for the calculated band structures.

### 2.5. Photocatalytic Activity of Eu-Doped ZnWO_4_ Nanoplates

The photocatalytic activity of Eu-doped ZnWO_4_ nanoplates was evaluated by monitoring the degradation of methyl orange in water under the irradiation from a high-pressure mercury lamp (100 W). The primary emission lines of the high-pressure mercury lamp were located at 365.0, 404.7, 435.8, and 546.1 nm. As described in previous work, the reactor consisted of a high-pressure mercury lamp, an inner cylindrical quartz tube (Φ55 mm), a middle cylindrical quartz tube (Φ75 mm), and an outer cylindrical quartz tube (Φ140 mm). The inner cylindrical quartz tube was designed to house the high-pressure mercury lamp. The free space between the inner and the middle cylindrical glass tubes served as the working chamber by filling 400 mL of the methyl orange solution for photocatalytic degradation. In the meanwhile, the free space between the middle and the outer cylindrical glass tubes was filled with running water to keep the temperature of the methyl orange solution lower than 40 °C. The height of each cylinder was 210 mm. The bottoms of the three co-axial cylinders were sealed together. In the present work, the concentration of methyl orange solution was about 56 mM (i.e., 18.3 mg/L). Detailed descriptions on the geometry of the photocatalytic reactor were available elsewhere [43,44]. After having been loaded with ZnWO_4_ nanoplates (400 mg), the solution of methyl orange was magnetically stirred in the dark for 30 min to ensure the establishment of an adsorption–desorption equilibrium. After having been exposed to the irradiation for a certain period of time, 5 mL of the suspension was collected. The particles of the photocatalysts were removed by centrifuging at 3000 rpm for 10 min before the absorbance measurement. The concentration of methyl orange was determined by checking the absorbance with an UV-vis spectrophotometer (UV2450, Shimazu, Japan).

### 2.6. Specific Surface Area and Chemical Oxygen Demand (COD) Measurements

The specific surface area of Eu^2+^ and Eu^3+^ co-doped ZnWO_4_ nanoplates was measured using a surface area analyzer (ASAP2010C, Micromeritics, Norcross, GA, USA) on the basis of nitrogen absorption at −196 °C. The samples were degassed overnight at 150 °C before nitrogen adsorption. The obtained nitrogen adsorption–desorption isotherms were evaluated with the Brunauer–Emmett–Teller (BET) equation to give the values of their specific surface areas. To confirm the complete mineralization of the dye, we derived the COD values at different stages of the degradation via the potassium dichromate titration method [45]. Dye solution sample (20 mL) was refluxed with HgSO_4_ (0.4 g), K_2_Cr_2_O_7_ (0.25 mol/L, 10 mL), and the mixture of AgSO_4_ and H_2_SO_4_ (5g AgSO_4_ in 500 mL H_2_SO_4_, 30 mL) at 150 °C for 2 h. Then, the dye solution was titrated with ferrous ammonium sulfate (0.1 mol/L) using ferroin indicator. A blank titration was carried out with deionized water. The equation for the COD value determination was described in the literature [45].

## 3. Results and Discussions

### 3.1. Morphology and Crystal Structure of Eu-Doped ZnWO_4_ Nanoplates

**Figure 1:**
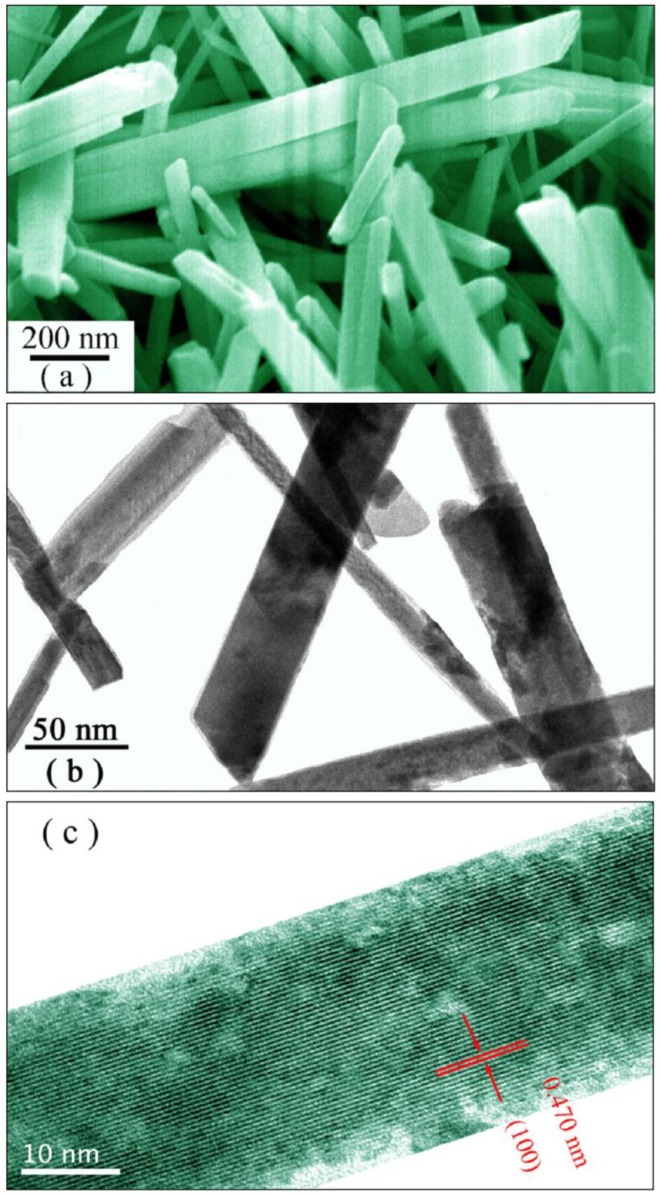
Micrographs of Eu-doped ZnWO_4_ nanoplates: (**a**) scanning electron microscopy (SEM) micrograph; (**b**) low-resolution transmission electron microscopy (TEM) micrograph; (**c**) high-resolution TEM micrograph.

Figure 1 shows the typical SEM micrograph (a), low-resolution TEM micrograph (b), and high-resolution TEM micrograph (c) of Eu-doped ZnWO_4_ nanoplates. The formation of ZnWO_4_ nanoplates is evident in Figure 1a. As can be seen in Figure 1a, the length of the ZnWO_4_ nanoplates is not uniform, and varies from 200 nm to several micrometers. Similarly, the width of the ZnWO_4_ nanoplates changes in the range of 20–80 nm. Moreover, the thickness of the nanoplates can be estimated from the SEM micrograph too, when their highly exposed facets are perpendicular to the paper plane, in which case, the nanoplates look apparently like nanorods. In this way, the thickness of ZnWO_4_ nanoplates is estimated to be around 10 nm. Further evidence on the formation of ZnWO_4_ nanoplates can be found in the low-resolution TEM micrograph. As shown in Figure 1b, the image contrast of each nanoplate is nearly uniform across the entire nanoplate. At lower magnifications, TEM image contrast is the result of differential absorption of electrons by the material, and the difference in thickness of the material will inevitably generate difference in TEM image contrast. If one ZnWO_4_ nanorod was the result, the TEM image contrast would be decreased from the edge of the nanorod towards its central axis because the thicker area will appear darker in a bright field image. This argument is confirmed by the darker image contrast when two ZnWO_4_ nanoplates are crossed over each other in Figure 1b. Thus, the uniform contrast in Figure 1b confirms the formation of ZnWO_4_ nanoplates. When compared with the Er^3+^ doped ZnWO_4_ single crystals [4], our ZnWO_4_ nanoplates are nanomaterials with large specific surface area. When compared with ZnWO_4_ nanoparticles and nanorods synthesized via the sol-gel, sonochemical, and hydrothermal methods [3,11,15,21,22], our Eu-doped ZnWO_4_ nanoplates are unique in their highly exposed facets. As documented in the literature, the exposed crystal facets directly determine their physicochemical properties [31,32,33,34]. Thus, acquiring a high percentage of reactive facets by crystal facet engineering is highly desirable for improving the photocatalytic reactivity of ZnWO_4_. In order to reveal the information of the facet, we performed high-resolution TEM characterization for the ZnWO_4_ nanoplates. As displayed in Figure 1c, the spacing between two adjacent planes is calculated to be 0.471 nm, which is in good agreement with the distance between two (100) crystal planes of ZnWO_4_. According to the results in Figure 1, our ZnWO_4_ nanoplates exhibit a highly exposed {100} facet. A similar TEM analysis shows that ZnWO_4_ nanoparticles have no obvious crystal orientation.

The formation of ZnWO_4_ nanoplates has something to do with the presence of CTAB in the hydrothermal reaction system. Hydrothermal synthesis is the technique of crystallizing substances from high-temperature aqueous solutions at high vapor pressures. The high temperature and high vapor pressure in the autoclave give the crystal a chance to develop into various kinds of crystal habits. CTAB, which is one of the most common surfactants, can lower the interfacial tension between two liquids or between a liquid and a solid. For instance, Ni et al. reported that the presence of CTAB can influence the growth orientation of ZnO under hydrothermal conditions [46]. In our case, one Zn^2+^ cation and one WO_4_^2−^ anion are turned into one ZnWO_4_ molecule when they encounter each other in the solution. Such ZnWO_4_ molecules stack together at the molecular scale to form a regular crystal lattice with lots of dangling bonds for further reaction. It is known that one CTAB molecule contains a hydrophobic group (tail) and a hydrophilic group (head). On the one hand, when a specific surface of the crystal lattice is not capped by the CTAB molecules, Zn^2+^ and WO_4_^2−^ ions from the aqueous solution can readily attach to this rough surface with the result of growing relatively quickly. On the other hand, when the surface is capped with the CTAB molecules on a molecular scale, Zn^2+^ and WO_4_^2−^ ions from the aqueous solution cannot so easily attach to this smooth surface for reactions, and hence this surface advances more slowly. As a result of the competitive growth, facets will appear on the growing ZnWO_4_ crystal because the CTAB adsorbed surface grows much more slowly than others. Figure 1 demonstrates that the ZnWO_4_ crystals grow very slowly in the <100> direction to allow the facets {100} to fully develop.

**Figure 2:**
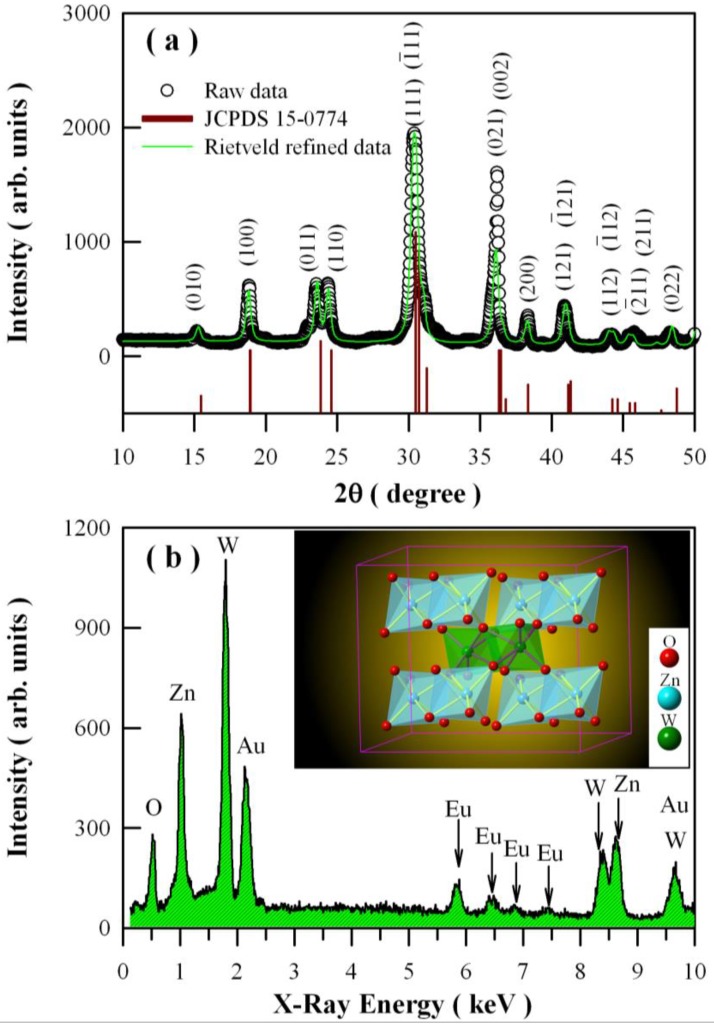
X-ray diffraction (XRD) curve (**a**) and energy dispersive X-ray (EDX) spectrum (**b**) of Eu-doped ZnWO_4_ nanoplates. Inset in (**b**): ZnO_6_ and WO_6_ octahedrons in ZnWO_4_.

Figure 2a gives the powder XRD curve of the Eu-doped ZnWO_4_ nanoplates. The open circles in Figure 2a represent the experimental data. As shown in Figure 2a, diffraction peaks at 14.98°, 18.86°, 23.56°, 24.36°, 38.30° and 48.36° can be assigned to the reflections from the (010), (100), (011), (110), (200), and (022) planes of monoclinic ZnWO_4_ [3,27], respectively, whereas the peak at 30.40° is ascribed to the combined contributions from (111), ($\bar{1}11$),and (020) crystallographic planes as these diffractions are located too closely [2,3,6,7,10,16]. For the same reason, the four peaks located at 36.20°, 40.94°, 44.22° and 45.76° can be ascribed to the contributions from the pairs of planes (021) and (002), (121) and ($\bar{1}21$), (112) and ($\bar{1}12$), and (211) and ($\bar{2}11$), respectively. The XRD data for standard ZnWO_4_ (Joint Committee on Powder Diffraction Standards (JCPDS), No. 15-0774) are depicted by the vertical bars in the bottom of Figure 2a for comparison. It can be seen that all the diffraction peaks of the sample can be readily indexed to the pure monoclinic phase ZnWO_4_. The solid green curve in Figure 2a represents the calculated diffractogram using the Rietveld refinement [40]. The lattice parameters obtained from the Rietveld refinement are *a* = 0.4683 nm, *b* = 0.5741 nm, *c* = 0.4949 nm, and *β* = 90.595°, which are nearly consistent with the standard data (*a* = 0.4691 nm, *b* = 0.5720 nm, *c* = 0.4925 nm, and *β* = 90.64°). Thus, the XRD curve in Figure 2a has verified that the ZnWO_4_ nanoplates are in monoclinic phase.

EDX is an analytical technique used for the elemental analysis of a specimen [42,43]. Figure 2b depicts the EDX spectrum of the Eu-doped ZnWO_4_. The first four X-ray emission peaks in the left section of Figure 2b are located at 0.53 keV, 1.02 keV, 1.78 keV, and 2.13 keV, which can be attributed to the characteristic X-ray emissions of O(Kα_1_), Zn(Lα_1,2_), W(Mα_1_), and Au(Mα_1_), respectively. The Au element was introduced in the specimen during the Au sputtering for the convenience of SEM analysis [47,48]. In the middle section of Figure 2b, there are four peaks located at 5.85 keV, 6.46 keV, 6.85 keV, and 7.48 keV, which can be assigned to the characteristic emissions of Eu(Lα_1,2_), Eu(Lβ_1_), Eu(Lβ_2,15_), and Eu(Lγ_1_), respectively [41,43]. In the right section of Figure 2b, the peak at 8.40 keV can be attributed to W(Lα_1_). In the meanwhile, the peak at 8.62 keV can be attributed to Zn(Kα_1_) and Zn(Kα_2_). It is interesting to note that the two characteristic emissions of Zn(Ka_1_) at 8.64 keV and Zn(Kα_2_) at 8.62 keV are merged into one peak at 8.64 keV because they are located near to each other. Because of a similar reason, the characteristic emissions of W(Lβ_1_) at 9.67 keV and Au(Kα_1_) at 9.71 keV are merged into one peak at 9.67 keV. As the characteristic emission peaks of Zn, O, W, and Eu are identified in the sample, we can conclude that the sample is primarily composed of Zn, O, W, and Eu. Without considering Au atoms in the sample, the atomic percentages of Zn, W, O, and Eu in Eu-doped ZnWO_4_ nanoplates are 25.9 at%, 22.0 at%, 48.3 at%, and 3.8 at%, respectively. For Eu-doped ZnWO_4_ nanoplates with the doping concentration of 5 mol %, the ideal atomic percentages of Zn, W, O, and Eu should be 16.53 at%, 16.53 at%, 66.11 at%, and 0.83 at%, respectively. It is obvious that the EDX technique can only give a rough quantification of Eu ions in ZnWO_4_ nanoplates.

The data in Figure 2 indicate that doping with Eu ions does not significantly modify the crystal structure of ZnWO_4_. It is known that the unit cell of monoclinic ZnWO_4_ is composed of two ZnWO_4_ molecules. Thus, one unit cell consists of two Zn sites, two W sites, and eight O sites. The inset in Figure 2b represents the ZnO_6_ and WO_6_ octahedrons formed in ZnWO_4_. In this structure, each W^6+^ ion is surrounded by six O ions with approximately octahedral coordination, and each Zn^2+^ ion is coordinated with six O ions to form an octahedron. All the metal-oxygen octahedra are distorted from perfect octahedral geometry. As can be seen in the inset, the structure of ZnWO_4_ is composed of zig-zag metal-oxygen chains made up of edge-sharing ZnO_6_ and WO_6_ octahedra [49]. Moreover, each (ZnO_6_–ZnO_6_)_n_ chain is interlinked to four chains of (WO_6_–WO_6_)_n_ and vice versa. As we know, the ionic radii of both Eu^3+^ (*r* = 94.7 pm) and Eu^2+^ (*r* = 117 pm) are close to that of Zn^2+^ (*r* = 90 pm when coordination number = 6) [50], but the six coordinated W^6+^ (*r* = 60 pm) sites are too small for Eu^2+^ or Eu^3+^ to occupy. Therefore, we believe that both Eu^2+^ and Eu^3+^ ions prefer to occupy the Zn^2+^ site in ZnWO_4_ nanoplates. Nominally, upon doping, there is an expansion of the lattice parameters to account for the enhanced atomic size of the dopant. However, no significant changes in the lattice parameters of the doped ZnWO_4_ samples are observed in Figure 2a. In our case, the concentration sum of Eu^2+^ and Eu^3+^ ions in ZnWO_4_ nanoplates is 5 mol %. As listed above, the ionic radius of Eu^3+^ is nearly equal to that of Zn^2+^, but the ionic radius of Eu^2+^ is about 30% more than that of Zn^2+^. So only Eu^2+^ ions can contribute significantly to the lattice expansion when its concentration is high enough. Our XPS analysis shows that the concentration of Eu^2+^ ions in ZnWO_4_ is only 1.7 mol %. That might be the reason that no obvious lattice expansion can be observed in Eu-doped ZnWO_4_ nanoplates.

### 3.2. XPS Spectra of Eu-Doped ZnWO_4_ Nanoplates

**Figure 3:**
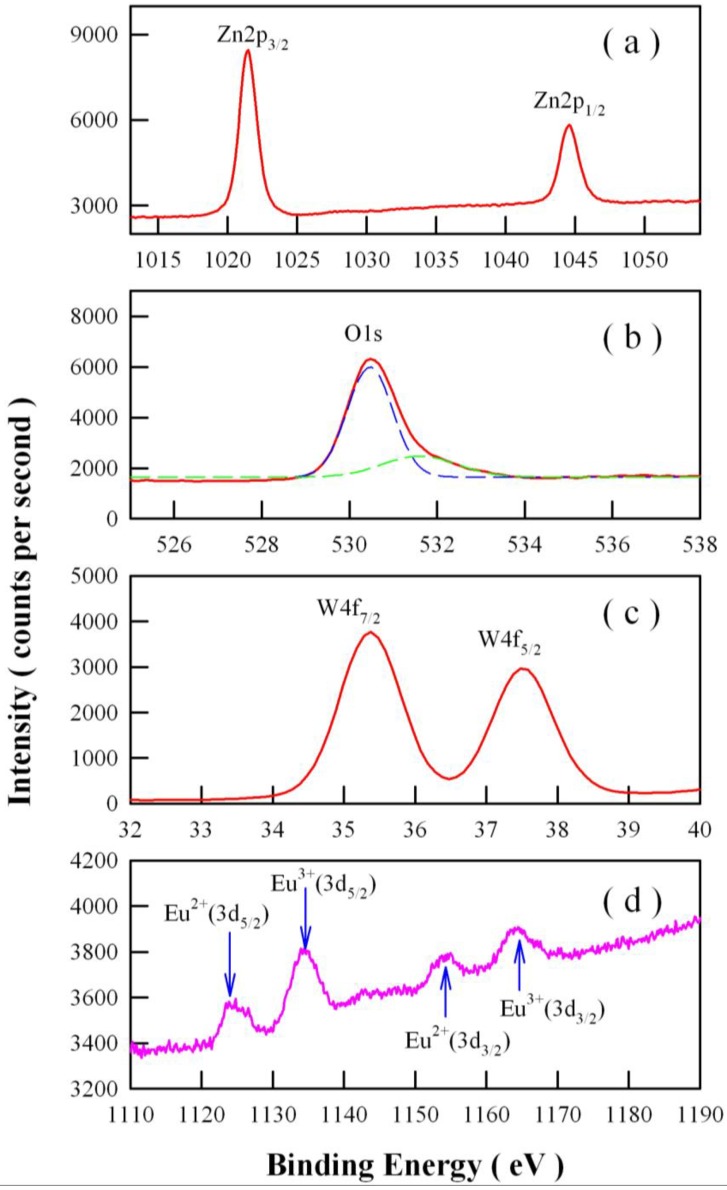
High-resolution X-ray photoelectron spectroscopic (XPS) spectra of Eu-doped ZnWO_4_ nanaplates: (**a**) Zn2p_3/2_ and Zn2p_1/2_; (**b**) O1s; (**c**) W4f_7/2_ and W4f_5/2_; (**d**) Eu3d_3/2_ and Eu3d_5/2_.

Ionic Eu is a well-known mixed-valence material whose valence state can be either Eu^3+^ or Eu^2+^ in various chemical environments [41,51,52]. Therefore, it is essential to examine the chemical states of Eu ions in ZnWO_4_ nanoplates. Figure 3 represents the high-resolution XPS spectra of Zn2p, O1s, W4f, and Eu3d in Eu-doped ZnWO_4_ nanoplates. It can seen in Figure 3a that the characteristic peaks of Zn2p_3/2_ and Zn2p_1/2_ are located at 1021.48 eV and 1044.58 eV, respectively. The separation between the two peaks is 23.1 eV, and the two peaks correspond to the typical Zn^2+^ oxidation states in ZnWO_4_ nanoplates [19,27]. As shown in Figure 3b, the XPS spectral profile of O1s is peaked at 530.48 eV, detailed analysis shows that an additional component appears at about 532 nm in the XPS spectrum of O1s. Oxides usually have oxygen vacancies. As is the case, an additional component in O1s line would show up. It is obvious that the XPS spectral profile of O1s can be decomposed into a component centered at 530.47 nm (dashed blue curve) and one component centered at 531.60 nm (dashed green curve). In actual fact, the shoulder at around 531.60 eV is related to the oxygen vacancies in ZnWO_4_. Figure 3c shows that the peaks of W4f_7/2_ and W4f_5/2_ are located at approximately 35.38 eV and 37.48 eV, respectively. The two peaks can be assigned to W4f_7/2_ and W4f_5/2_ signals and are consistent with the W^6+^ in ZnWO_4_ [19,27,53]. Unexpectedly, we recorded the characteristic XPS peaks of mixed states of Eu in the ZnWO_4_ nanoplates. As shown in Figure 3d, four XPS peaks can be clearly identified at 1126.4 eV, 1134.6 eV, 1155.7 eV, and 1163.2 eV. It is known that the Eu3d_5/2_ core levels of Eu^2+^ and Eu^3+^ ions in the XPS spectra are clearly different from each other in energy positions, as are the Eu3d_3/2_ core levels of Eu^2+^ and Eu^3+^ ions in their XPS spectra. Thus, the first two peaks in Figure 3d can be assigned to Eu^2+^ (3d_5/2_) and Eu^3+^ (3d_5/2_) core-levels, while the last two peaks in Figure 3d can be assigned to Eu^2+^ (3d_3/2_) and Eu^3+^ (3d_3/2_) core-levels, respectively [51,52]. The binding energy of Eu^2+^(3d_5/2_) is 29.3 eV lower than that of Eu^2+^(3d_5/2_), and the binding energy of Eu^3+^(3d_5/2_) is 28.6 eV lower than that of Eu^3+^(3d_3/2_). The area ratios of the XPS signals are approximately 1.32:2.43:1.00:1.47 for Eu^2+^(3d_5/2_)/Eu^3+^(3d_5/2_)/Eu^2+^(3d_3/2_)/Eu^3+^(3d_3/2_). Employing a standard of Eu^2+^ doped ZnWO_4_ with the doping concentration of 1 mol % as reference, we measured its high-resolution XPS spectrum of Eu3d_3/2_ and Eu3d_5/2_. The peak areas of Eu^2+^ (3d_5/2_) at 1126.4 eV and Eu^2+^ (3d_3/2_) at 1155.7 eV are obtained by integration of the spectrum. It is assumed that the area of a peak is proportional to the total amount of Eu^2+^ species responsible for the peak. This results in a direct relation between the peak area fraction and the mole fraction of Eu^2+^ species in the sample. By comparing the peak areas of Eu^2+^ (3d_3/2_) and Eu^2+^ (3d_5/2_) in Figure 3d with those of the standard sample, we can determine the doping percentage of Eu^2+^ in the Eu-doped ZnWO_4_ nanoplates. In this way, the doping percentage of Eu^2+^ in Eu-doped ZnWO_4_ was derived to be around 1.7 mol %, meaning the doping percentage of Eu^3+^ in Eu-doped ZnWO_4_ was about 3.3 mol %. Consequently, the data in Figure 3d have pointed out the coexistence of Eu^2^^+^ and Eu^3+^ in ZnWO_4_ nanoplates, although Eu^3+^ ions were the only doping source in the starting materials. The reason of the coexistence of Eu^2^^+^ and Eu^3+^ in ZnWO_4_ nanoplates is that a fraction of Eu^3+^ ions are self-reduced to Eu^2+^ ions during the growth of nanocrystals, as we discussed for the case of Eu-doped SrSO_4_ [39].

**Figure 4:**
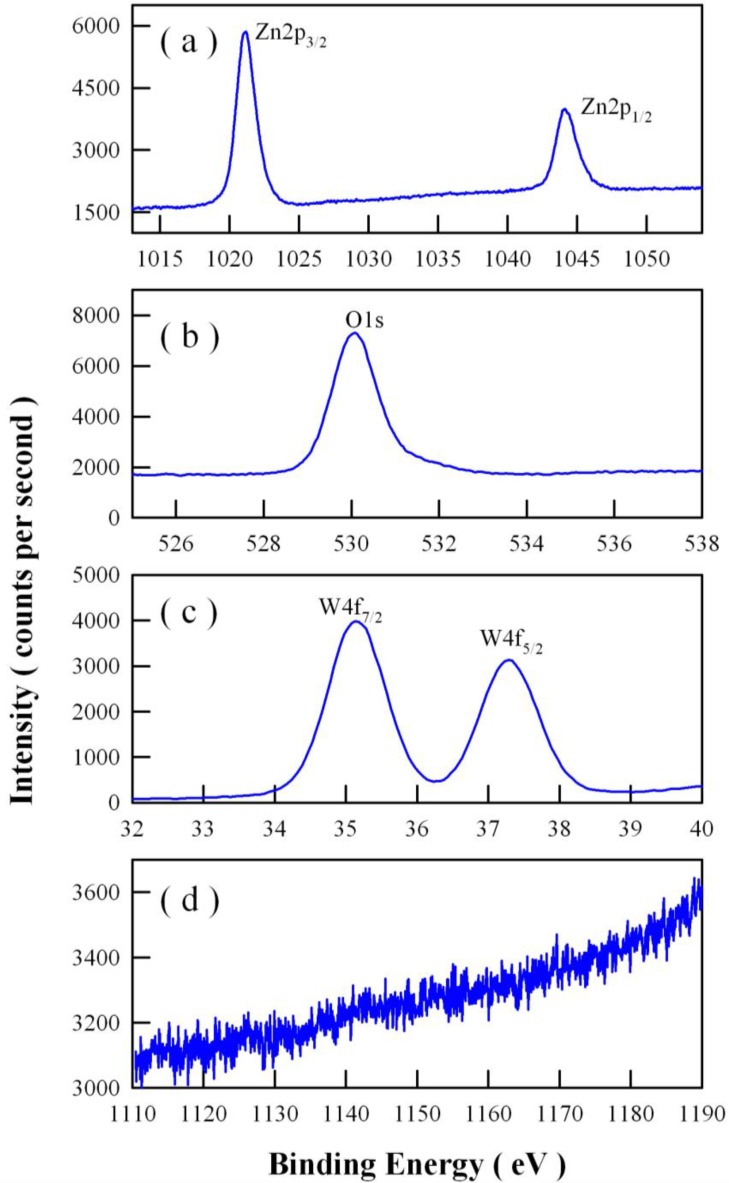
High-resolution XPS spectra of undoped ZnWO_4_ nanaplates: (**a**) Zn2p_3/2_ and Zn2p_1/2_; (**b**) O1s; (**c**) W4f_7/2_ and W4f_5/2_; (**d**) Eu3d_3/2_ and Eu3d_5/2_.

The XPS spectra of undoped ZnWO_4_ nanoplates are provided so that variation of the energy levels can be seen much more clearly by the readers. Figure 4 displays the high-resolution XPS spectra of Zn2p, O1s, W4f, and Eu3d in undoped ZnWO_4_ nanoplates. As shown in Figure 4a, the peaks of Zn2p_3/2_ and Zn2p_1/2_ are located at 1021.16 eV and 1044.13 eV, respectively. When compared with the peaks of Zn2p_3/2_ (1021.48 eV) and Zn2p_1/2_ (1044.58 eV) in Eu-doped ZnWO_4_ nanoplates, the two peaks are shifted 0.32 and 0.45 eV, respectively, towards the lower binding energy. In Figure 4b, the XPS spectral profile of O1s is located at 530.08 eV, which is 0.40 eV lower in binding energy than that of O1s in Eu-doped ZnWO_4_ nanoplates (530.48 eV). Similarly, the peaks of W4f_7/2_ and W4f_5/2_ in Figure 4c are located at 35.13 eV and 37.28 eV, which are 0.25 eV and 0.20 eV lower in binding energy than those in Eu-doped ZnWO_4_ nanoplates (35.38 and 37.48 eV), respectively. Obviously, no Eu-related peaks appear in Figure 4d, indicating the absence of Eu in the undoped ZnWO_4_ nanoplates. Consequently, the data in Figure 4 demonstrate that the binding energies of Zn2p, O1s, and W4f in Eu-doped ZnWO_4_ nanoplates are higher than those in undoped ZnWO_4_ nanoplates. These chemical shifts indicate that doping ZnWO_4_ with Eu ions has generated noticeable changes in the local bonding environment around Zn, O, and W sites. Additionally, XPS can also be employed to study the electronic surface states and band bending of Eu-doped ZnWO_4_ nanoplates. Because of the termination of lattice periodicity at the surfaces of ZnWO_4_ nanoplates, the unpaired electrons in the dangling bonds of surface atoms interact with each other to form an electronic state with a narrow energy band in the semiconductor band gap. Obviously, these surface states are determined by the atomic structure of the semiconductor surface. Once these surface states are present, they can induce band bending for ZnWO_4_ nanoplates. The effects of band bending on photochemistry and photocatalysis are discussed in some reviews.

The presence of Eu^2+^ ion in ZnWO_4_ indicates that some Eu^3+^ ions in ZnWO_4_ are reduced to Eu^2+^ ions in the process of crystal growth. It is known that oxygen vacancy (V_O_) can be easily produced in the lattice of ZnWO_4_ in the crystal growth phase. As one V_O_ is formed in ZnWO_4_, one positively charged V_O_ is left in the lattice. In the meanwhile, one negatively charged oxygen species is released into the lattice in order to keep the lattice neutral. When the negatively charged oxygen species diffuses randomly in the lattice, it donates its electrons with the liberation of oxygen out of the lattice. This process can be described by Equation (1):

(1) $$2O^{-}-2e\to O_{2}$$

In this way, the vacancy V_O_ would act as a donor of electrons. Eu^3+^ ion can be reduced to Eu^2+^ by capturing the released electron. This process can be described by Equation (2):

(2) $$Eu^{3+}+e\to Eu^{2+}$$

A detailed discussion on the self-reduction of Eu^3+^ to Eu^2+^ can be found elsewhere [39].

### 3.3. Absorption and PL Spectra of Eu-Doped ZnWO_4_ Nanoplates

**Figure 5:**
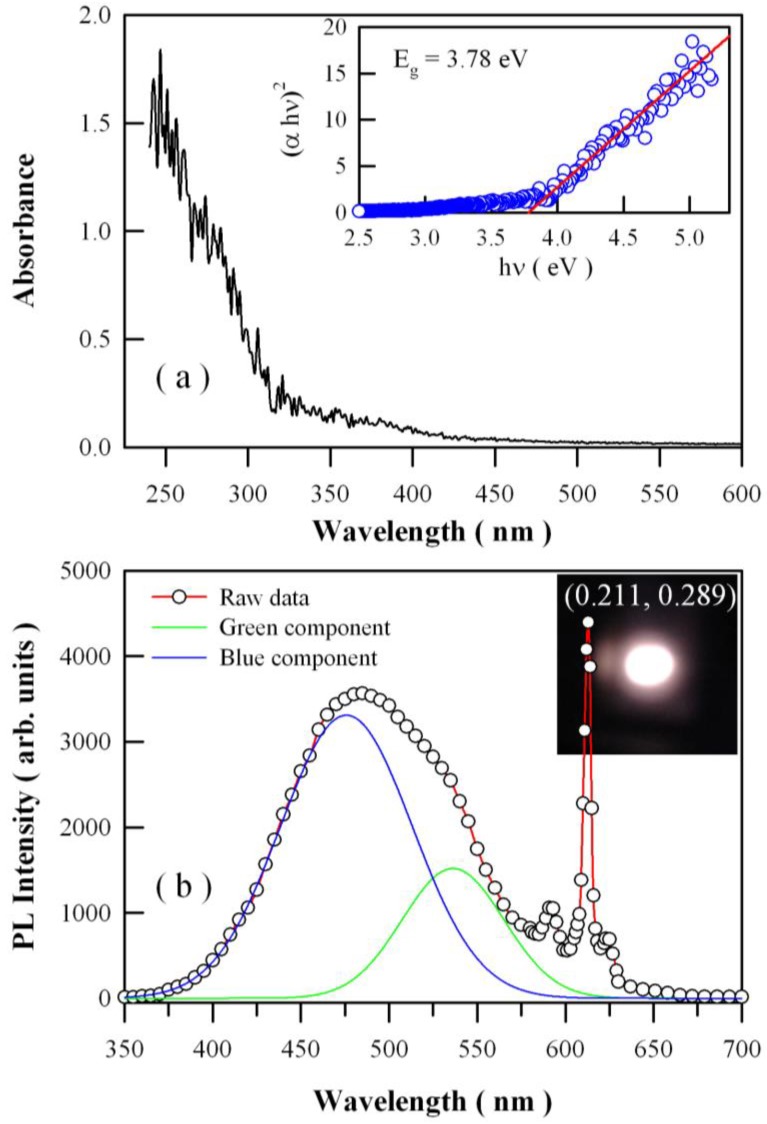
(**a**) UV-vis absorption spectrum of Eu-doped ZnWO_4_ nanoplates; (**b**) photoluminescence (PL) spectrum of Eu-doped ZnWO_4_ nanoplates. Inset in (**b**): luminescence photo of Eu-doped ZnWO_4_.

It is known that both the absorption and density of defects are important factors to determine the photocatalytic activity of a photocatalyst [54,55,56]. Figure 5a shows the absorption spectrum of the Eu-doped ZnWO_4_ nanoplates. It can be seen that this absorption spectrum can be divided into two sections. The first section ranges from 240 nm to 310 nm, while the second section ranges from 310 nm to 450 nm. When compared with the absorption spectra of single crystal ZnWO_4_ [57], we can assign the first absorption band to the band-edge absorption of ZnWO_4_ nanoplates, while the second absorption band to defects in Eu-doped ZnWO_4_ nanoplates. According to the first principles calculations by Kalinko et al., monoclinic ZnWO_4_ crystals is a direct semiconductor [58]. Thus, the bandgap value of Eu-doped ZnWO_4_ nanoplates can be calculated from the Tauc plot. As depicted by the inset in Figure 5a, the bandgap value of Eu-doped ZnWO_4_ nanoplates is equal to 3.78 eV. By measuring the diffuse reflectance spectrum of ZnWO_4_ film coated on quartz substrate, Zhao et al. reported that the experimental bandgap of the ZnWO_4_ film was about 4.01 eV [8]. It can be seen that our derived bandgap value roughly agrees with that reported by Zhao et al.

Figure 5b represents the PL spectrum of Eu-doped ZnWO_4_ nanoplates. The hollow blue circles in Figure 5b represent the experimental PL data. It is obvious that the Eu-doped ZnWO_4_ nanoplates exhibit a broadband emissions centered at around 487 nm and two sharp emissions at 592 nm and 612 nm. At a first glance, the broad PL band can be decomposed into two Guassian bands with their peaks centered at 475.8 nm (2.61 eV) and 536.2 nm (2.31 eV), which are shown by the solid blue curve and the solid green curve, respectively, in Figure 5b. ZnWO_4_ generally exhibits a broad blue-green emission band with its peak at about 480 nm (2.6 eV) [1], and this PL band is often attributed to a charge transfer between oxygen and tungsten ions in the [WO_6_]^6−^ molecular complex [1,57]. However, such assignment is quite elusive for physicists. In the view of solid state physics, the origins of PL can be classified into band edge emission and defect emission. It is known that defects are important structural features to dominate the PL properties in a variety of metal oxides [41,59,60]. This also holds true for ZnWO_4_ nanoplates, where coordinatively unsaturated vacancies are active sites for luminescence. Due to the large difference between its bandgap (about 4 eV) and its emission energy (around 2.6 eV), we can exclude the possibility of band edge recombination as the candidate of the greenish blue PL of ZnWO_4_ nanoplates. This feature allows us to assign the broadband PL to certain kinds of defects in ZnWO_4_ nanoplates. Intrinsic defects such as O, W, and Zn vacancies are likely candidates of the luminescence centers.

As for the two sharp emissions at 592 nm and 612 nm in Figure 5b, it becomes quite straightforward to assign them to the electronic transitions ^5^D_0_→^7^F_1_ and ^5^D_0_→^7^F_2_ of Eu^3+^ ions in the host matrix of ZnWO_4_ [39,41,61,62]. As is well known, the ^5^D_0_→^7^F_1_ line originates from magnetic dipole transition, while the ^5^D_0_→^7^F_2_ line results from the electric dipole transition. In terms of the Judd–Ofelt theory, the magnetic dipole transition is permitted, but the electric dipole transition is allowed only on condition that the Eu ion occupies a site without an inversion center. The results in Figure 5b indicate that most of Eu^3+^ ions do not occupy the inversion center sites in ZnWO_4_. The lack of inversion symmetry and the break of parity selection rules in ZnWO_4_ make the ^5^D_0_→^7^F_2_ electric dipole transition the strongest among all the transitions. As a result of Eu^2+^ and Eu^3+^ codoping, both the defect density and the PL properties of ZnWO_4_ nanoplates can be effectively modified. With the method as described in previous work [48,59,63], the CIE chromaticity coordinates of the Eu-doped ZnWO_4_ nanoplates are determined to be (0.211, 0.289), and the correlated color temperature is derived to be 19000 K for the greenish blue PL.

### 3.4. Electronic Structures of Perfect ZnWO_4_ and Defect-Containing ZnWO_4_

**Figure 6:**
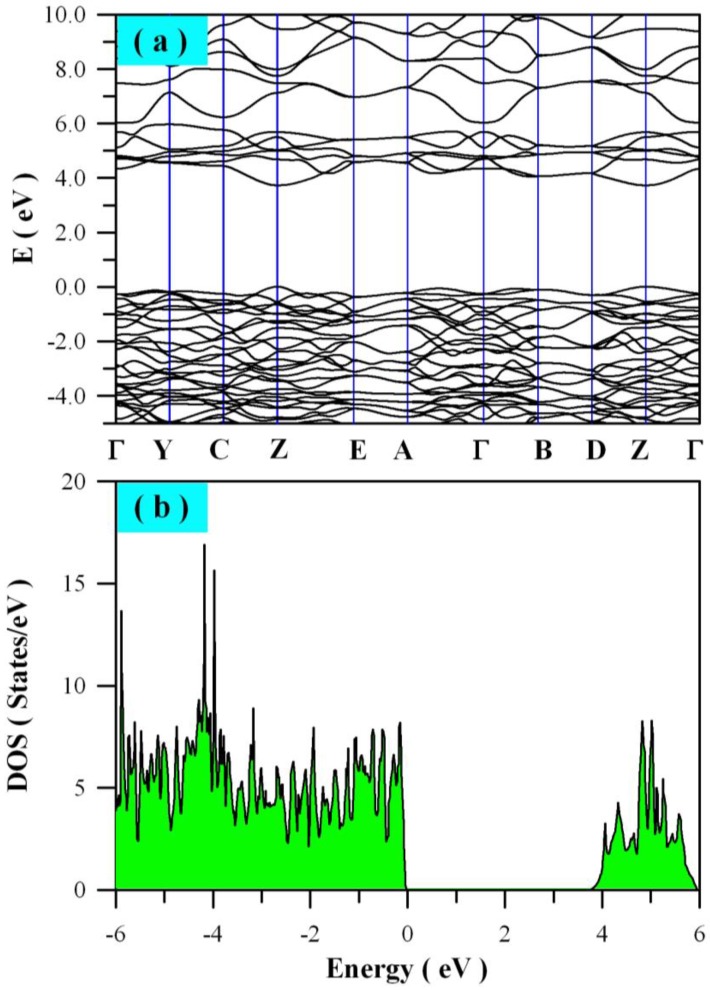
Electronic structures of perfect ZnWO_4_: (**a**) band structures; (**b**) density of states (DOS).

The electronic structure of a photocatalyst is not only critically important to understand its absorption and luminescent properties, but also the most important factor to determine its photocatalytic activity. Density functional calculations can be reliably applied to electronic structure calculations for a variety of materials [39,47,48,59]. In the framework of GGA + U, we performed electronic structure calculations for perfect ZnWO_4_ and defect-containing ZnWO_4_ by defining U^5d^ = 8 eV for W. Figure 6 presents the calculated band structures and density of states of perfect ZnWO_4_. It can be seen that some bands at the bottom of conduction band (CB) are not flat, which is the typical character of a semiconductor. As shown in Figure 6a, both the maximum of valence band (VB) and the minimum of CB are located at Z point, confirming that ZnWO_4_ is a semiconductor with direct bandgap. Figure 6b depicts the density of states of defect-free ZnWO_4_. It is clear that the bandgap of ZnWO_4_ is free of any impurity energy levels. Using the LDA approach, Kalinko et al. reported that ZnWO_4_ is direct semiconductor with its bandgap value of around 2.31 eV [58]. It is obvious that our calculated bandgap value (3.72 eV) is much closer to the experimental value (about 4.0 eV) when compared with Kalinko’s data.

**Figure 7:**
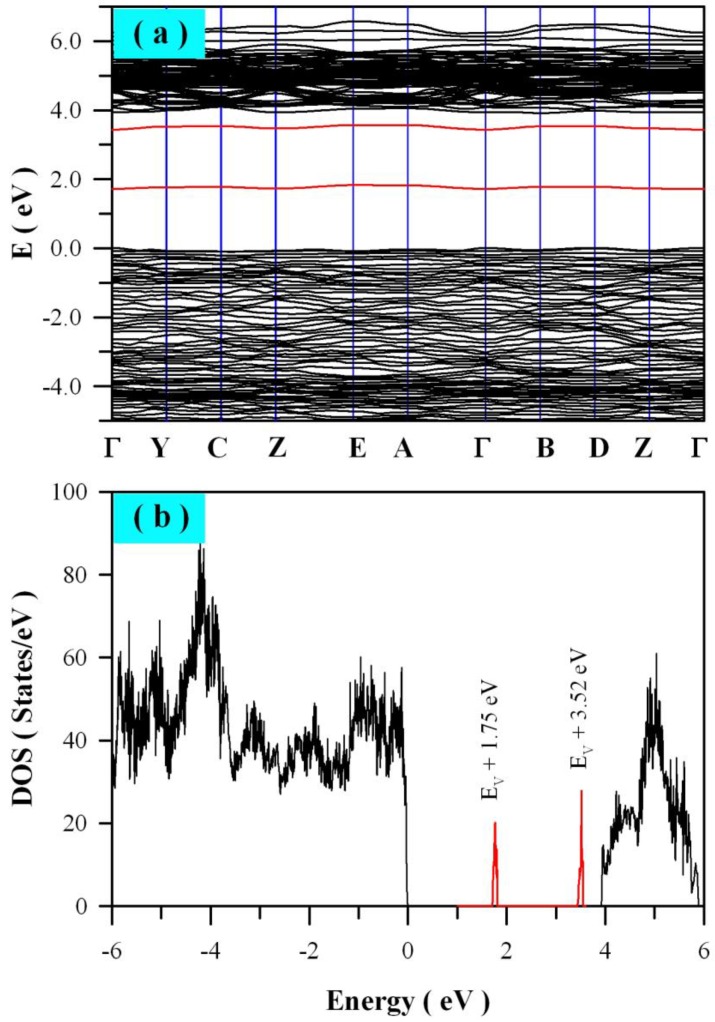
Electronic structures of ZnWO_4_ bearing 1 mol % of oxygen vacancy: (**a**) band structures; (**b**) density of states.

V_O_ is one of the most fundamental defects in ZnWO_4_, and it influences many physical properties of the material such as charge trapping and recombination. Therefore, detailed knowledge of the electronic structures of V_O_ is essential in understanding the PL of ZnWO_4_ nanoplates. In order to model V_O_ in ZnWO_4_, we built a 2 × 2 × 2 super cell that contains 64 O sites, 16 W sites, and 16 Zn sites. After one O site was removed from the super cell, ZnWO_4_ with around 1 at% of V_O_ was the result. Figure 7 represents the calculated band structures and density of states of V_O_ bearing ZnWO_4_. As shown in Figure 7a, the calculated bandgap of V_O_ bearing ZnWO_4_ is 3.91 eV when U^5d^ = 8 eV for W. When compared with the bandgap of perfect ZnWO_4_, the V_O_ bearing ZnWO_4_ exhibits a bandgap that is a little bit wider (ca. 0.19 eV). The most prominent feature in Figure 7 is that V_O_ can introduce two defect energy levels in the bandgap of ZnWO_4_, one of which is located at E_V_ + 1.75 eV, while the other is located at E_V_ + 3.52 eV. The two defect energy levels can be clearly identified in Figure 7b, where the V_O_ introduced defect energy levels are marked in red. As it is positively charged, V_O_ can act as electron trap sites as well as luminescence centers [64].

**Figure 8:**
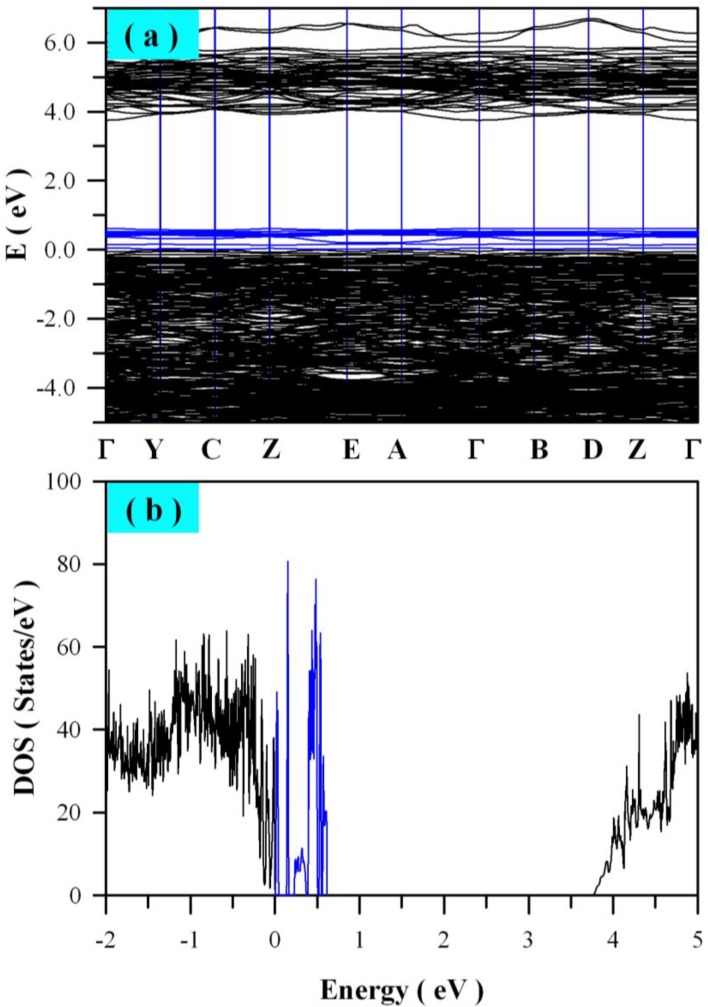
Electronic structures of ZnWO_4_ bearing 1 mol % of W vacancy: (**a**) band structures; (**b**) density of states.

Besides V_O_, we further considered tungsten vacancy (V_W_) and zinc vacancy (V_Zn_) in ZnWO_4_. Figure 8 shows the calculated band structures and density of states of V_W_ bearing ZnWO_4_. As can be seen in Figure 8a, the bandgap remains direct with the value of 3.75 eV. The defect energy levels introduced by V_W_ are located in the range from VB to E_V_ + 0.62 eV. Additionally, we calculated the band structures and density of states for V_Zn_ bearing ZnWO_4_. It is found that the defect energy levels of V_Zn_ are located in the bandgap of ZnWO_4_, but they are very close to the edge of VB (<0.1 eV). For the sake of clarity, we do not present here the calculated band structures and density of states for V_Zn_ bearing ZnWO_4_.

Belonging to *4f–5d* transition, Eu^2+^ doped nanomaterials generally show a broad PL band ranging from ultraviolet through visible to infrared region. However, Eu^2+^ doped ZnWO_4_ nanoplates exhibit no extra PL band in the visible range when compared with undoped ZnWO_4_ nanoplates. As for Eu^3+^ doped ZnWO_4_, the *4f* electrons of the dopant are sufficiently localized to form multiple atomic-like states in the band gap of ZnWO_4_ due to the shielded *4f*-shell. As the DFT calculations are a one-electron theory, the DFT with GGA scheme fails to accurately predict the multi-electron properties for Eu^3+^ ions in ZnWO_4_. Consequently, neither the energies of *J* multiplets of Eu^3+^ ions (^7^F_J_, where J = 0–6) in the ground state nor the energy levels in the excited state of Eu^3+^ ions (^5^D_0_) can be deduced correctly from the DFT calculations. That is why we did not model the defect energy levels of Eu^3+^ ZnWO_4_. Fortunately, both the energy levels of Eu^3+^ in the ground state and in the excited state vary by only a small amount in different hosts. Thus, the energy levels of ^7^F_J_ and ^5^D_0_ of Eu^3+^ in ZnWO_4_ can be determined by making use of the experimentally obtained energies for Eu^3+^, except that the exact location of the lowest energy level of Eu^3+^ is unknown for the case of ZnWO_4_.

### 3.5. Time-Resolved PL Spectra and Possible PL Mechanism of Eu-Doped ZnWO_4_ Nanoplates

**Figure 9:**
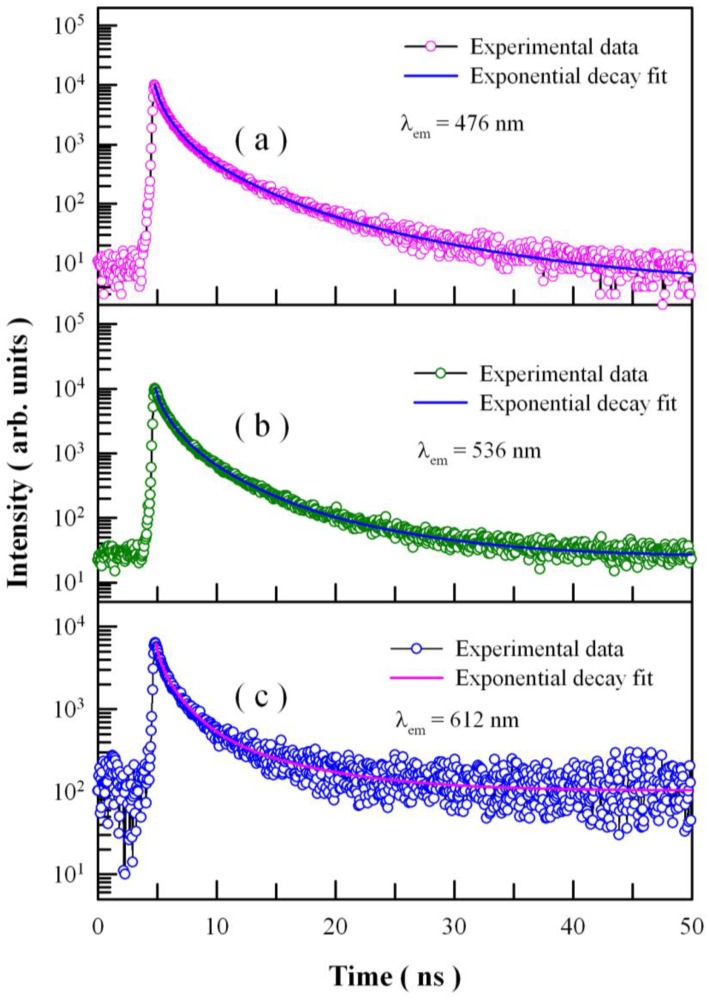
Time-resolved PL spectra of Eu-doped ZnWO_4_ nanoplates taken at different emission wavelengths: (**a**) 476 nm; (**b**) 536 nm; (**c**) 612 nm.

More physical insight could be gained by studying the time-resolved PL behaviors of Eu-doped ZnWO_4_ nanoplates [65]. Figure 9 depicts the time-resolved PL spectra of Eu-doped ZnWO_4_ nanoplates with the emission wavelength fixed at 476 nm, 536 nm, and 612 nm, respectively. The excitation wavelength is 375 nm. Circles in Figure 9 represent the experiment data, and the solid lines represent the fitted curves. It is found that each decay curve in Figure 9 can be fitted with quadruple exponential function as show in Equation (3):

(3) $$I(t)=A_{0}+A_{1}\exp\left(-\frac{t}{\tau_{1}}\right)+A_{2}\exp\left(-\frac{t}{\tau_{2}}\right)+A_{3}\exp\left(-\frac{t}{\tau_{3}}\right)+A_{4}\exp\left(-\frac{t}{\tau_{4}}\right)$$

where *I*(*t*) refers to the PL intensity at time *t*, *A*_0_ is the baseline, *A_i_* is the *i*th pre-exponential factor of the decay components, and *τ_i_* is the *i*th decay time component (*i* = 1–4). The fitting parameters of the time-resolved PL spectra are listed in Table 1. The parameter χ^2^ in Table 1 represents the goodness of fit, and the average lifetime <*τ*> is calculated using the following Equation (4) [66]:

(4) $$\langle \tau \rangle =\frac{A_{1}\tau_{1}^{2}+A_{2}\tau_{2}^{2}+A_{3}\tau_{3}^{2}+A_{4}\tau_{4}^{2}}{A_{1}\tau_{1}+A_{2}\tau_{2}+A_{3}\tau_{3}+A_{4}\tau_{4}}$$

These parameters bear important information on the kinetics of carrier recombination. For example, in the case of Figure 9a, the decay time constants *τ*_1_ = 0.30 ns, *τ*_2_ = 1.04 ns, *τ*_3_ = 3.21 ns, *τ*_4_ = 9.73 ns, and <*τ*> = 3.041 ns for the PL emission at 476 nm. It is noted that *τ*_1_ is at the limit of the measurement capability of the instrument, and therefore it merely represents the order of the short decay time constant [40,60,65]. The coexistence of *τ*_2_, *τ*_3_, and *τ*_4_ suggests the presence of three kinds of luminescence centers in Eu-doped ZnWO_4_ nanoplates. As discussed in Figure 4, Figure 5, Figure 6 and Figure 7, the three luminescence centers in Eu-doped ZnWO_4_ nanoplates are correlated to one extrinsic defect Eu^3+^ and two intrinsic defects V_O_ and V_W_.

A careful analysis of the lifetime constants provides an understanding of the local environment around the luminescence centers in Eu-doped ZnWO_4_ nanoplates. As listed in Table 1, the average lifetime of Eu-doped ZnWO_4_ nanoplates is in the range of 3–4 ns, which is about 1000 times shorter than the long PL lifetime (3.9 μs) of ZnWO_4_ single crystals grown by the Czochralski method [67]. When compared with ZnWO_4_ single crystal, Eu-doped ZnWO_4_ nanoplates are characteristic of a large number of surface defects because of their large surface area. The shortened lifetime in Eu-doped ZnWO_4_ nanoplates can be attributed to the non-radiative relaxation produced by a large number of surface defects that act as quenching centers. Additionally, Wang et al. reported that the PL lifetimes of ZnWO_4_ nanoparticles were about 100 ns [68]. It is obvious that the average lifetime of Eu-doped ZnWO_4_ nanoplates is about 30 times shorter than the PL lifetime of undoped ZnWO_4_ nanoparticles. This is understandable because codoping with Eu^2+^ and Eu^3+^ inevitably provides extra non-radiative recombination paths in Eu-doped ZnWO_4_ nanoplates. Finally, we have noticed that the average lifetime increases from 3.041 ns to 3.745 ns as the monitoring wavelength increases from 476 nm to 612 nm. The increase in the PL lifetime at a longer emission wavelength reflects the changes in the micro-environments (i.e., non-radiative recombination paths) around the blue, green, and red luminescence centers.

**Figure 10:**
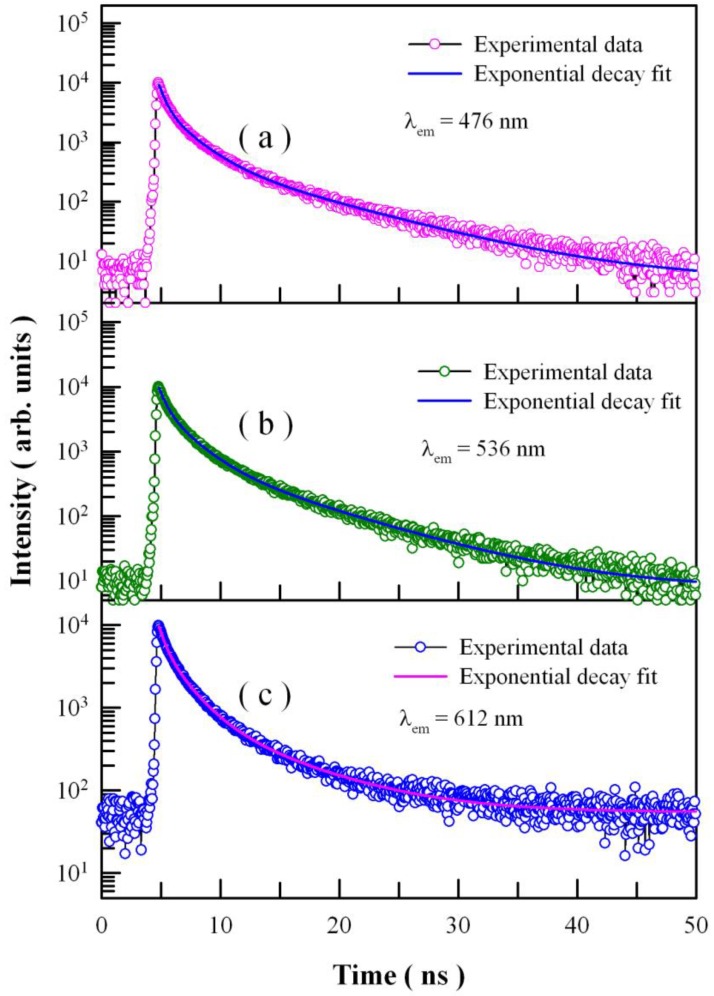
Time-resolved PL spectra of undoped ZnWO_4_ nanoplates taken at different emission wavelengths: (**a**) 476 nm; (**b**) 536 nm; (**c**) 612 nm.

Figure 10 shows the time-resolved PL spectra of undoped ZnWO_4_ nanoplates with the emission wavelength fixed at 476 nm, 536 nm, and 612 nm, respectively. The excitation wavelength is 375 nm. Circles in Figure 10 represent the experiment data, and the solid lines represent the fitted curves. It is found that each decay curve in Figure 10 can be fitted with a triple exponential function. The fitting parameters of the time-resolved PL spectra are listed in Table 1. When compared with the data for undoped ZnWO_4_ nanoplates, Eu-doped ZnWO_4_ nanoplates exhibit longer average lifetimes at the detection wavelengths of 476 nm and 536 nm. This is understandable because the defects introduced by Eu-doping generate extra recombination paths, which in turn shorten the lifetimes of the blue and green bands.

**Figure 11:**
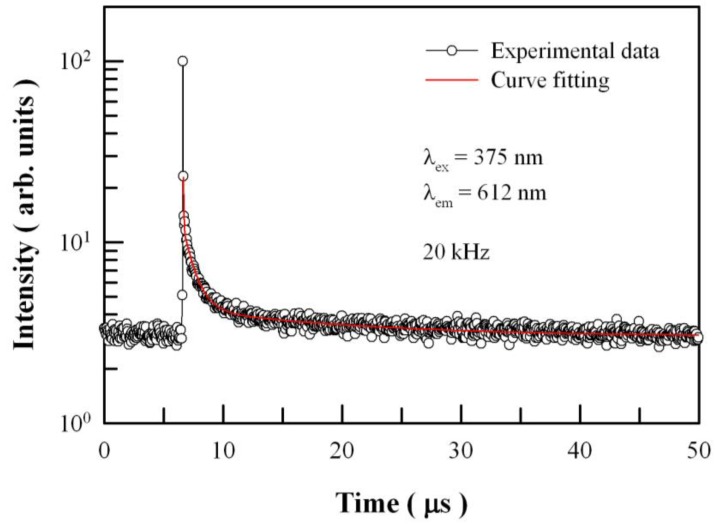
Time-resolved PL spectrum of Eu-doped ZnWO_4_ nanoplates at emission wavelength of 612 nm. The repetition frequency of the pulsed laser diode is 20 kHz.

It is very strange to get lifetimes in the order of nanoseconds for Eu emission lines. They usually have lifetimes much greater, even milliseconds. In addition, it is uncommon that both defect and Eu emissions have similar lifetimes. It is found that the critical point rests on the repetition frequency of the picosecond pulsed diode laser. The repetition frequency of the picosecond pulsed diode laser was 20 MHz in the lifetime measurements for Figure 9 and Figure 10. The pulse period associated with this repetition frequency is only 50 ns, which is not long enough to measure the lifetime ranging from microsecond to millisecond. In order to measure the lifetime of Eu emissions at 612 nm, we have to employ a pulsed diode laser with much longer pulse period by decreasing its repetition rate to 20 kHz. The repetition rate of 20 kHz implies 50 ms between two pulses. Figure 11 shows the time-resolved PL spectrum of Eu-doped ZnWO_4_ nanoplates at the emission wavelength of 612 nm. The repetition frequency of the pulsed laser diode is 20 kHz. This decay curve can be fitted with triple exponential function with the fitting parameters of t_1_ = 40.78 ns, t_2_ = 963.25 ns, and t_3_ = 13956.67 ns. The pre-exponential factors are *A*_1_ = 36.50, *A*_2_ = 8.01, and *A*_3_ = 1.26. Indeed, the average lifetime of Eu^3+^ emissions is calculated to be 9.455 ms. As documented in the literature, Wang et al. reported that the average lifetime of Pr^3+^ doped ZnWO_4_ at 607 nm was 5.40 ms [66]. It is clear that the average lifetimes of Eu^3+^ emissions and Pr^3+^ emissions in ZnWO_4_ are at the same order of magnitude. Moreover, we can see that the average lifetime of Eu^3+^ emissions is about three orders of magnitude larger than those of the intrinsic defect emissions in ZnWO_4_ nanoplates.

**Figure 12:**
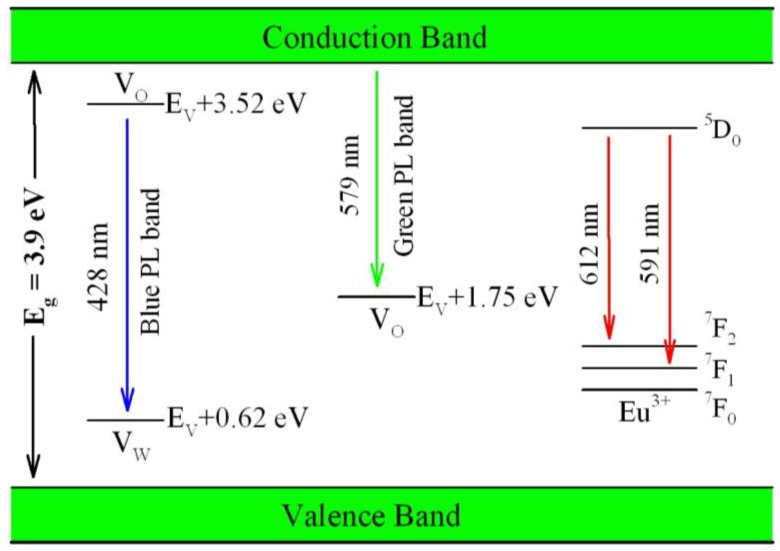
Possible mechanisms of the defect related emissions in Eu-doped ZnWO_4_.

Figure 12 illustrates the possible mechanism of defect related emissions in Eu-doped ZnWO_4_. As displayed in Figure 12, the bandgap value of ZnWO_4_ is assumed to be 3.9 eV, the V_O_ introduced defect energy levels are located at E_V_ + 1.75 eV and E_V_ + 3.52 eV, while V_W_ introduced defect energy level is located at E_V_ + 0.62 eV. Under the UV excitation, the first kind of radiative recombination is the electrons trapped by V_O_ at the defect energy level E_V_ + 3.52 eV to recombine with the holes trapped by V_W_ at the defect energy level E_V_ + 0.62 eV. Such a kind of radiative recombination leads to the blue PL band peaking at around 428 nm (2.90 eV). The second kind of radiative recombination is the electrons in CB to recombine with the holes trapped by the V_O_ at E_V_ + 1.75 eV. Such a kind of radiative recombination yields the green PL band peaking at around 577 nm (2.15 eV). The third kind of radiative recombination is the electrons in the excited state ^5^D_0_ of Eu^3+^ to recombine radiatively with the holes in its ground states ^7^F_1,2_, leading to the characteristic emissions peaking at 591 nm and 612 nm. When compared with the blue PL band peaking at 475.8 nm (2.61 eV) and the green PL band peaking at 536.2 nm (2.31 eV) in Figure 5b, our predicted emission energies of the defect-related emissions in ZnWO_4_ roughly agree with the actual ones. The differences between the predicted emission energies and the actual emission energies rest on the fact that it is hard to exactly and reliably determine the defect energy levels with DFT calculation after having considered the limitations in semi-local approximations to DFT [39].

### 3.6. Specific Surface Area of Eu-Doped ZnWO_4_ Nanoplates

**Figure 13:**
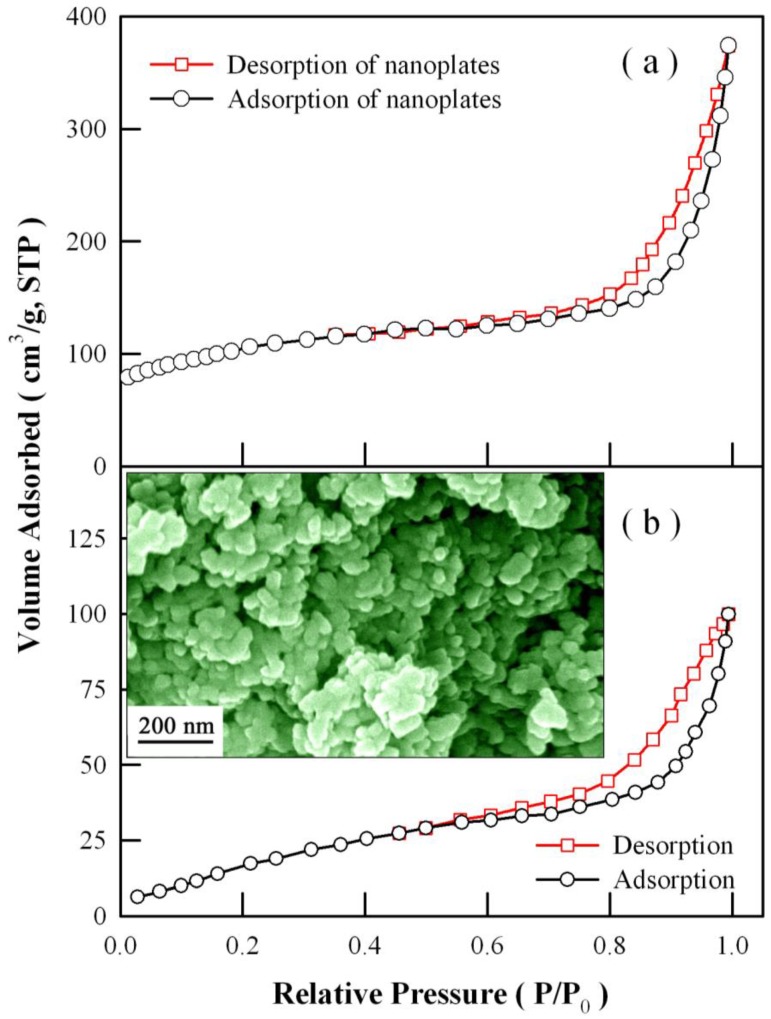
(**a**) Nitrogen adsorption and desorption isotherms of Eu-doped ZnWO_4_ nanoplates; (**b**) nitrogen adsorption and desorption isotherms of undoped ZnWO_4_ nanoparticles. Inset in (**b**): SEM micrograph of the undoped ZnWO_4_ nanoparticles.

The photocatalytic activity of a photocatalyst is generally complicated by a number of factors such as the light absorption capacity, the specific surface area, the grain size, the defect density, and so on. [43,44,54,56] Among these factors, the surface area of a photocatalyst is one of the key factors to influence its photocatalytic activity. BET theory aims to explain the physical adsorption of gas molecules on a solid surface and serves as basis for an important analysis technique to measure the specific area of materials. To obtain the information about the specific surface area of the Eu-doped ZnWO_4_ nanoplates, we performed BET nitrogen adsorption isotherm measurements at 77 K on a Micrometrics ASAP 2010. Figure 13a depicts the typical nitrogen adsorption and desorption isotherms of Eu-doped ZnWO_4_ nanoplates. As shown in Figure 13a, the nitrogen adsorption isotherm belongs to type II, and the specific surface area of Eu-doped ZnWO_4_ nanoplates is derived to be 344 m^2^/g. Figure 13b represents the typical nitrogen adsorption and desorption isotherms of undoped ZnWO_4_ nanoparticles. The inset in Figure 13b depicts the SEM micrograph of the undoped ZnWO_4_ nanoparticles. Data analysis shows that the specific surface area of the undoped ZnWO_4_ nanoparticles is about 79.4 m^2^/g. The experimental results revealed that the specific surface area of ZnWO_4_ nanoplates is higher than that of ZnWO_4_ nanoparticles. The lower surface area of ZnWO_4_ nanoparticles may be caused by the aggregation of ZnWO_4_ nanoparticles. As shown by the inset of Figure 13b, ZnWO_4_ nanoparticles are easily aggregated. Such an aggregation leads to the dramatic reduction in the specific surface area of ZnWO_4_ nanoparticles. In contrast, the Eu-doped ZnWO_4_ nanoplates are not easily aggregated because of their specific architectures. As documented in the literature, Yan et al. reported that the specific surface area of ZnWO_4_ nanocrystals was in the range of 25–28 m^2^/g [10]; Liu et al. determined the specific surface area of B-doped ZnWO_4_ nanorods to be 22–47.2 m^2^/g [24]; and Su et al. reported that the specific surface area was 109.4 m^2^/g for Sn^2+^-doped ZnWO_4_ nanocrystals [27]. It can also be seen that the specific surface area of our Eu-doped ZnWO_4_ nanoplates is much larger than the B-doped ZnWO_4_ nanorods, as well as Sn^2+^-doped ZnWO_4_ nanocrystals. Considering the fact that photocatalytic reactions mainly occur on the catalyst surface, the large surface area is helpful for gaining high photocatalytic activity for E-doped ZnWO_4_ nanoplates.

### 3.7. Photocatalytic Activity of ZnWO_4_ Nanoplates

**Figure 14:**
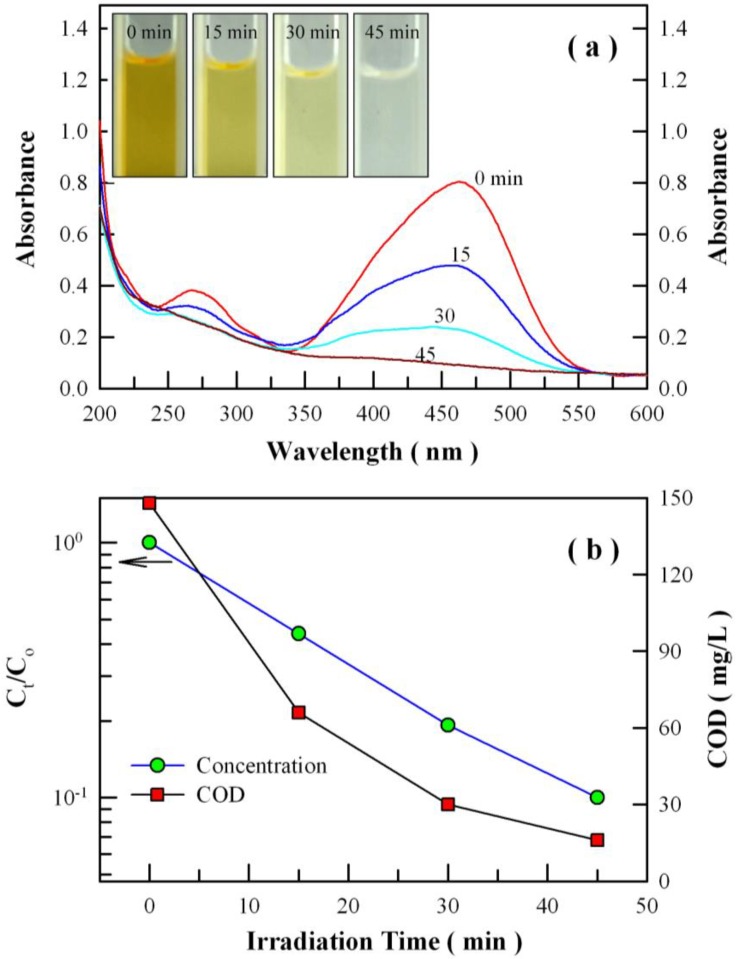
(**a**) Evolution of the absorption spectrum of methyl orange solution with the irradiation time of the high-pressure mercury lamp in the presence of Eu-doped ZnWO_4_ nanoplates; (**b**) semi-log plot of C_t_/C_0_ (circles) and chemical oxygen demand (COD) plot (squares) as a function of irradiation time of the high-pressure mercury lamp. Inset in (**a**): photos of methyl orange solutions at different stages of photocatalytic degradation.

Figure 14a displays the evolution of absorption spectrum of methyl orange solution with the irradiation time of the high-pressure mercury lamp in the presence of Eu-doped ZnWO_4_ nanoplates. It is clear that the methyl orange exhibits a strong absorption at about 463 nm and a weak absorption at about 268 nm. As documented in the literature, the strong absorption at 463 nm can be attributed to the large conjugation system in the methyl orange molecule, which is primarily comprised of the two phenyl chromophores and the azo linkage (–N=N–). The weak absorption at 268 nm can be attributed to the small conjugation system in the methyl orange molecule, which is comprised of the phenyl chromophore [54,55,56,69,70,71]. This assignment is evidenced by the absorptions of phenyl and its derivatives. For example, benzene (C_6_H_6_) exhibits absorption at 254 nm, tulene (C_6_H_5_CH_3_) exhibits absorption at 261 nm, phenol (C_6_H_5_OH) exhibits absorption at about 270 nm, and phenylanine (C_6_H_5_NH_2_) exhibits absorption at about 280 nm. The most prominent feature in Figure 14a is that both the absorption bands are decreased gradually upon UV irradiation until they disappear completely after the UV irradiation for 45 min. Thus, the simultaneous disappearance of the two absorption bands indicates that both the large and the small conjugation systems in the methyl orange are destroyed. It has been established that the photocatalytic degradation of organics in solution is initiated by the photoexcitation of the semiconductor, followed by the formation of an electron-hole pair on the surface of the ZnWO_4_ as shown in Equation (5) [54,55,56]. On one hand, very reactive hydroxyl radical can also be formed either by the decomposition of water (Equation (6)) or by the reaction of the hole with OH^−^ (Equation (7)).

(5) $$ZnWO_{4}+hv\to h_{VB}^{+}+e_{CB}^{–}$$

(6) $$h_{VB}^{+}+H_{2}O\to H^{+}+•OH$$

(7) $$h_{VB}^{+}+OH^{–}\to •OH$$

On the other hand, electron in the conduction band of the catalyst can reduce molecular oxygen to superoxide anion (Equation (8)).

(8) $$e_{CB}^{–}+O_{2}\to •O_{2}^{–}$$

Ultimately, the hydroxyl radicals are generated in both reactions. These hydroxyl radicals are very oxidative and non selective with redox potential of E_0_ = 2.8 V to oxidize organic compounds into fragments [49]. That is why the the Eu-doped ZnWO4 nanoplates are active photocatalysts.

The Langmuir–Hinshelwood kinetic model is widely used to describe the kinetics of photocatalytic degradation of many organic compounds. This model can be simplified to a pseudo first-order expression when the concentration of reagent being reacted is very low.

(9) $$C_{t}=C_{0}\exp\left(-kt\right)$$

where *C*_0_ is the initial concentration of dye, *C_t_* is the concentration of dye at instant *t*, and *k* is the pseudo first-order kinetic rate constant [20]. The photocatalytic kinetic rate constant for the methyl orange degradation can be determined using Equation (9). Figure 14b shows the semi-logarithmic plots of *C_t_*/*C*_0_ of the methyl orange solution versus the irradiation time of the high-pressure mercury lamp in the presence of Eu-doped ZnWO_4_ nanoplates (solid circles). The solid blue line in Figure 14b represents the curve fitting of the data with Equation (9). The semi-log plot of dye concentration versus time was linear, suggesting the first-order reactions for the photocatalytic degradation. In our case, the first-order kinetic rate constant of the photocatalytic reaction was derived to be 0.0542 min^−1^ for Eu-doped ZnWO_4_ nanoplates. To confirm the complete mineralization of the dye, we analyzed the COD values at different stages of the photocatalytic degradation. As shown by the solid squares in Figure 14b, the COD value is 148 for the methyl orange solution just before UV irradiation, but the COD values were decreased to 66, 30, and 16 after photocatalytic degradation for 15 min, 30 min, and 45 min, respectively. The dramatic decrease in the COD value suggests that the dye can be completely mineralized into CO_2_ and H_2_O by Eu-doped ZnWO_4_ nanoplates. These results have demonstrated that the Eu-doped ZnWO_4_ nanoplates exhibit superior photo-oxidative capability to completely mineralize methyl orange into CO_2_ and H_2_O.

**Figure 15:**
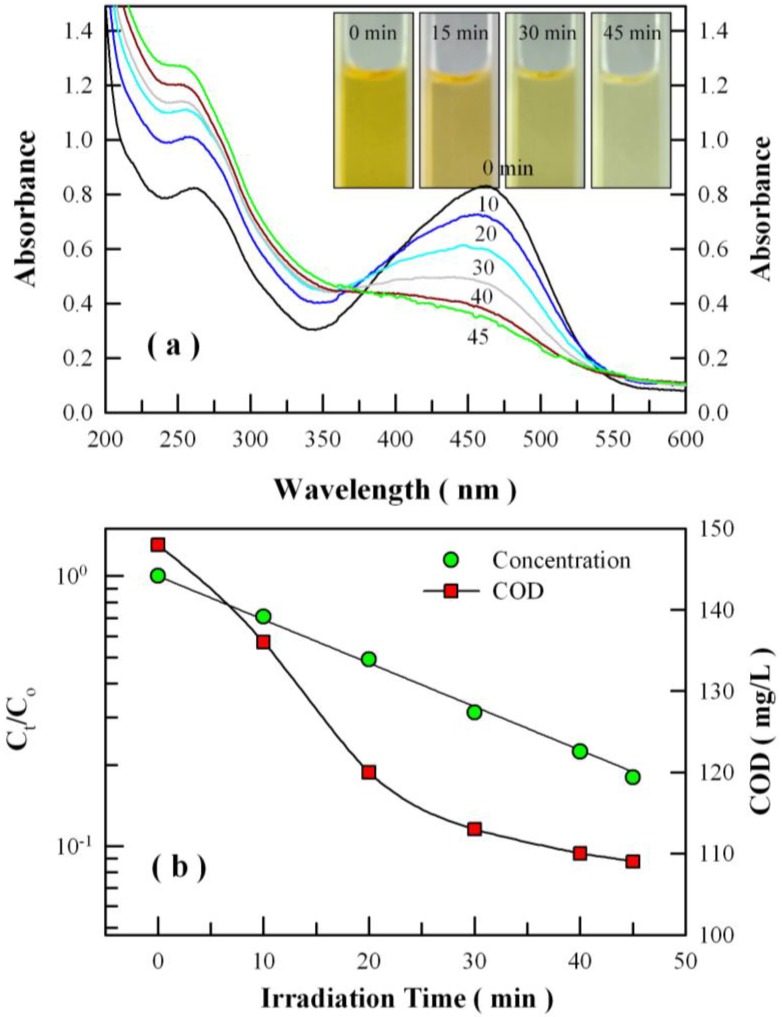
(**a**) Evolution of the absorption spectrum of methyl orange solution with the irradiation time of the high-pressure mercury lamp in the presence of undoped ZnWO_4_ nanoparticles; (**b**) semi-log plot of C_t_/C_0_ (circles) and COD plot (squares) as a function of irradiation time of the high-pressure mercury lamp. Inset in (**a**): photos of methyl orange solutions at different stages of photocatalytic degradation.

Figure 15a represents the evolution of absorption spectrum of methyl orange solution with the irradiation time of the high-pressure mercury lamp in the presence of undoped ZnWO_4_ nanoparticles. As the UV irradiation continues, the absorption band at 463 nm decreases at a much slower rate than in the case of Eu-doped ZnWO_4_ nanoplates. Figure 15b shows the semi-logarithmic plots of C_t_/C_0_ of the methyl orange solution versus the irradiation time of the high-pressure mercury lamp in the presence of undoped ZnWO_4_ nanoparticles (solid circles). The solid black line in Figure 14b represents the curve fitting of the data with Equation (9). The first-order kinetic rate constant of the photocatalytic degradation is derived to be 0.0371 min^−1^. When compared with Eu-doped ZnWO_4_ nanoplates, the photocatalytic activity of ZnWO_4_ nanoparticles is obviously lower than that of Eu-doped ZnWO_4_ nanoplates. The higher photocatalytic activity of of Eu-doped ZnWO_4_ nanoplates can be partially attributed to their large surface area. Doping with Eu^2+^ and Eu^3+^ ions is another factor enhancing the photocatalytic activity of Eu-doped ZnWO_4_ nanoplates. Positive effects of doping on the photocatalytic activity of ZnWO_4_ nanostructures were also reported, examples include non-metal ions (B, C, N, F) doping [24,25,26], transition metal ions doping (Sn^2+^, Cr^3+^, Mn^2+^, and Cu^2+^) [27,28], and rare-earth metal ions doping (Dy^3+^ and Er^3+^) [29,30]. Here, a cooperative mechanism involving both doping and surface area is believed to account for the higher photocatalytic activity of Eu-doped ZnWO_4_ nanoplates.

We have noticed that the weak absorption band in Figure 15a does not decrease significantly upon the UV irradiation. Moreover, the overall absorption in the range of 200–350 nm becomes stronger as the UV irradiation gets longer. This exotic feature in the absorption spectrum indicates the profound difference in the photocatalytic degradation behaviors between the undoped ZnWO_4_ nanoparticles and the Eu-doped ZnWO_4_ nanoplates. To check if the undoped ZnWO_4_ nanoparticles can completely mineralize the organics into H_2_O and CO_2_, we performed the COD analysis for the methyl orange solutions at different stages of photocatalytic degradation, and the derived COD data are shown in Figure 15b. Instead of dropping in a large scale to around 0, the COD value of the solutions only drops marginally from 148 to 109 as the UV irradiation extends from 0 min to 45 min. Such a marginal decrease in COD suggests that the dye molecules are cleaved into intermediates during the photocatalytic process of the undoped ZnWO_4_ nanoparticles.

With respect to undoped ZnWO_4_ nanoparticles, Eu-doped doped ZnWO_4_ nanoplates exhibit superior photocatalytic performance for dye degradations because they can completely mineralize the organic molecules into H_2_O and CO_2_, whereas undoped ZnWO_4_ nanoparticles can break the organic molecules into fragments only. Such a superior photocatalytic performance can be attributed to the highly exposed {100} facets of ZnWO_4_ nanoplates. It is known that the process of heterogeneous photocatalysis with semiconductor–based photocatalyst involves three mechanistic steps: the excitation, bulk diffusion, and surface transfer of photoexcited electrons and holes [31]. Apparently, the reactivity of ZnWO_4_ is definitely affected by surface atomic structures because surface atomic arrangement and coordination intrinsically determine the adsorption of reactant molecules, the surface transfer between photoexcited electrons and reactant molecules, and the desorption of product molecules. Consequently, the reactivity of ZnWO_4_ sensitively varies with crystal facets. That is the reason that Eu-doped ZnWO_4_ nanoplates with highly exposed facets can have dramatically different photocatalytic performance than ZnWO_4_ nanoparticles. Our results on the facet-dependent photocatalytic activity provide a feasible route to fabricating efficient ZnWO_4_ photocatalysts via crystal facet engineering.

**Figure 16:**
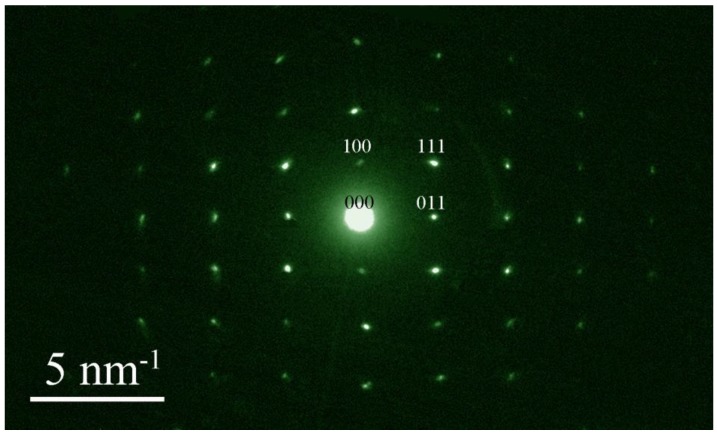
Selected area electron diffraction pattern of Eu-doped ZnWO_4_.

In order to elaborate the roles of crystal facets in the photocatalysis, we performed similar photocatalytic tests on undoped ZnWO_4_ nanoplates, as well as Eu-doped ZnWO_4_ nanoparticles. It is found that undoped ZnWO_4_ nanoplates can also completely mineralize the organic molecules into H_2_O and CO_2_, but at a slower decomposition rate than Eu-doped ZnWO_4_ nanoplates. In contrast, Eu-doped ZnWO_4_ nanoparticles can only break the organic molecules into fragments, although they can exhibit higher decomposition rates than undoped ZnWO_4_ nanoparticles. These results give additional evidence on the different roles played by crystal facets and rare-earth doping in the photocatalysis. Additionally, it is essential to properly identify the orientation of the plates, so we should provide the selected area electron diffraction pattern of Eu-doped ZnWO_4_ nanoplates. Figure 16 shows the selected area electron diffraction pattern of Eu-doped ZnWO_4_ nanoplates. It is clear that the zone axis of the nanoplates is [001].

## 4. Conclusions

Eu-doped ZnWO_4_ nanoplates with highly exposed {100} facets were synthesized via the CTAB assisted hydrothermal growth at 180 °C. Under the 325 nm laser excitation, the PL spectrum of Eu-doped ZnWO_4_ nanoplates consists of a broadband centered at around 487 nm and two sharp bands peaking at 592 nm and 612 nm. First-principles DFT calculations have been performed to provide insight onto the defect-related emissions of ZnWO_4_ nanoplates. It is found that Eu-doped ZnWO_4_ nanoplates exhibit superior photo-oxidative capability to completely mineralize methyl orange into H_2_O and CO_2_, whereas ZnWO_4_ nanoparticles can cleave methyl orange molecules into fragments only. The superior photocatalytic performance of ZnWO_4_ nanoplates rests on the fact that ZnWO_4_ nanoplates have highly exposed {100} facets, whereas ZnWO_4_ nanoparticles have no obvious facets. The study highlights the importance of crystal facets in photocatalytic systems and illustrates how crystal facet engineering can be utilized in combination with defect engineering to design novel photocatalytic materials with superior photo-oxidative capability.

## Figures and Tables

**Table 1:** Fitting parameters of the time-resolved photoluminescence (PL) spectra measured at different emission wavelengths (λ_em_) for Eu-doped ZnWO_4_ nanoplates and undoped ZnWO_4_ nanoplates.

	Eu-Doped ZnWO_4_ Nanoplates			Undoped ZnWO_4_ Nanoplates		
	λ_em_ = 476 nm	λ_em_ = 536 nm	λ_em_ = 612 nm	λ_em_ = 476 nm	λ_em_ = 536 nm	λ_em_ = 612 nm
*A*_0_	4.778	25.099	103.36	4.890	7.62	54.263
*A*_1_	4217.94	3064.32	1846.81	5977.83	5271.15	4510.53
*A*_2_	4896.78	5131.95	1606.39	3248.57	3969.98	4639.88
*A*_3_	1613.72	2284.13	2552.76	592.19	822.49	956.61
*A*_4_	196.35	401.56	578.93			
*τ*_1_ (ns)	0.30	0.26	0.28	0.54	0.59	0.55
*τ*_2_ (ns)	1.04	0.99	0.65	2.14	2.11	1.87
*τ*_3_ (ns)	3.21	2.94	1.83	8.02	7.58	6.63
*τ*_4_ (ns)	9.73	8.34	7.13			
<t> (ns)	3.04	3.32	3.75	3.66	3.77	3.41
χ^2^	1.123	1.101	1.122	1.110	1.171	1.076
